# V-Y advancement flap technique for severe cicatricial lower eyelid ectropion: A case report and outcome assessment

**DOI:** 10.1016/j.jpra.2025.10.020

**Published:** 2025-10-19

**Authors:** Donghao Yu, Zeming Zhao, Jianhua Xu

**Affiliations:** a Department of Ophthalmology, The Second Hospital of Hebei Medical University, Shijiazhuang, Hebei Province 050000, China; b Department of General Surgery, The Second Hospital of Hebei Medical University, Shijiazhuang, Hebei Province 050000, China; c Department of Ophthalmology, The Fourth People’s Hospital of Shenyang, Shenyang, Liaoning Province 050000, China

**Keywords:** V-Y advancement flap, Cicatricial ectropion, Scar contracture, Eyelid reconstruction, Tension redistribution, Minimally invasive surgery, Aesthetic outcomes, Functional restoration, Postoperative follow-up, patient satisfaction

## Abstract

A 63-year-old male with severe cicatricial lower eyelid ectropion was treated with the V-Y advancement flap technique. This method alters lateral tension to counteract excessive longitudinal tension, effectively correcting ectropion caused by scar traction. The V-Y technique not only simplifies surgical incision design with minimal risk of flap failure but also demonstrates the efficiency and flexibility of tension redistribution. Compared to full-thickness skin grafts or other flap techniques, it provides superior aesthetics and a more natural appearance. This technique serves as a valuable alternative and expands treatment options for cicatricial lower eyelid ectropion.

## Case report

**Figure 1:**
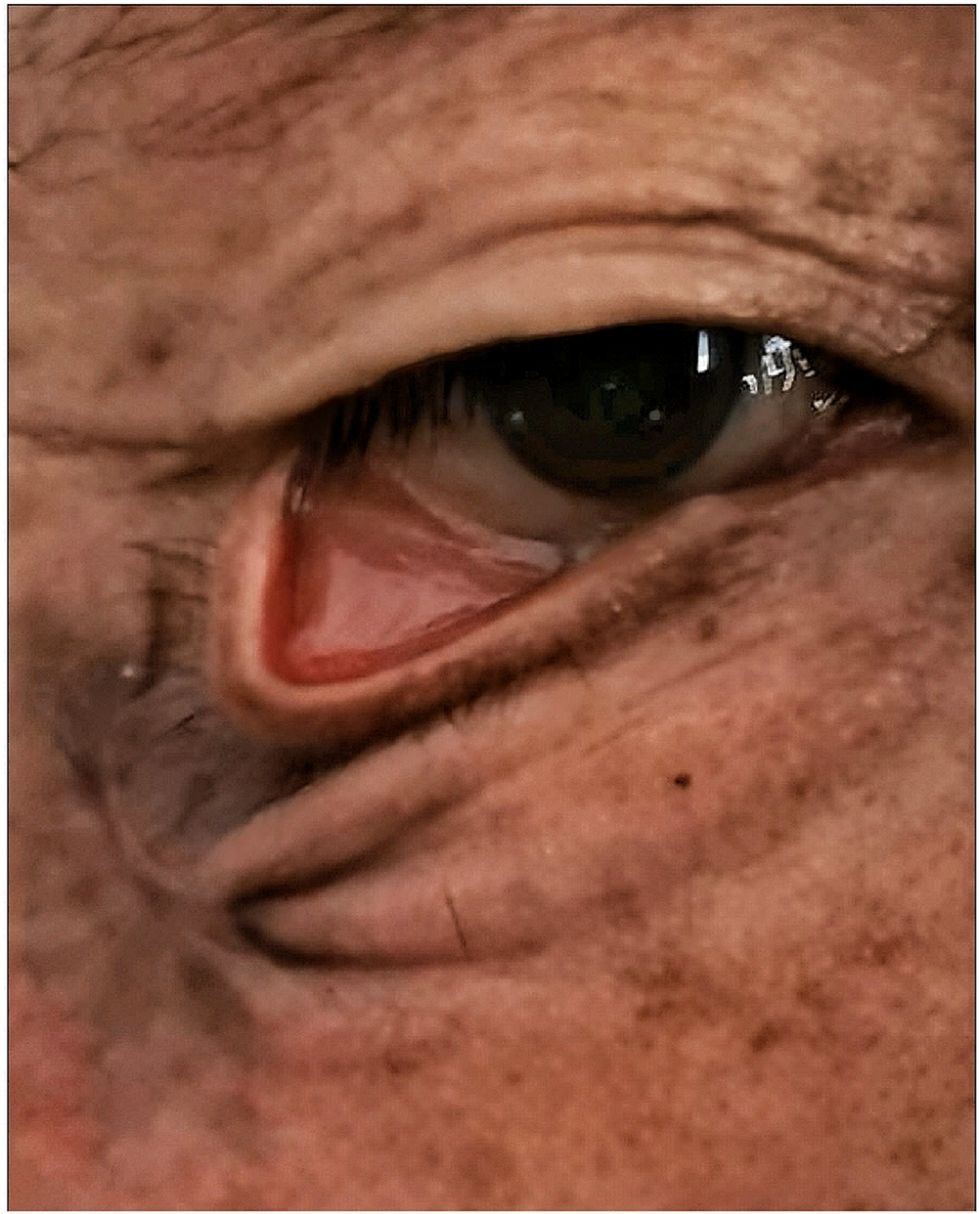
Presentation at our department: The wound had healed, but severe lower eyelid ectropion with three-layered skin folds was evident at the site of maximal scar contracture.

A 63-year-old male patient sustained a 2 × 2 cm skin defect on the lateral aspect of the right zygomatic arch due to a car accident . The wound was primarily sutured at a local clinic. It is worth noting that the patient subsequently developed mild lower eyelid ectropion, with a distinct separate scar from the primary closure located 0.5 cm below the eyelid margin . Due to a strong aversion to surgical skin grafts, the patient opted for conservative treatment with a powdered drug to promote wound healing . Three months later, he presented to our department with severe lower eyelid ectropion (Figure 1).

**Figure 2:**
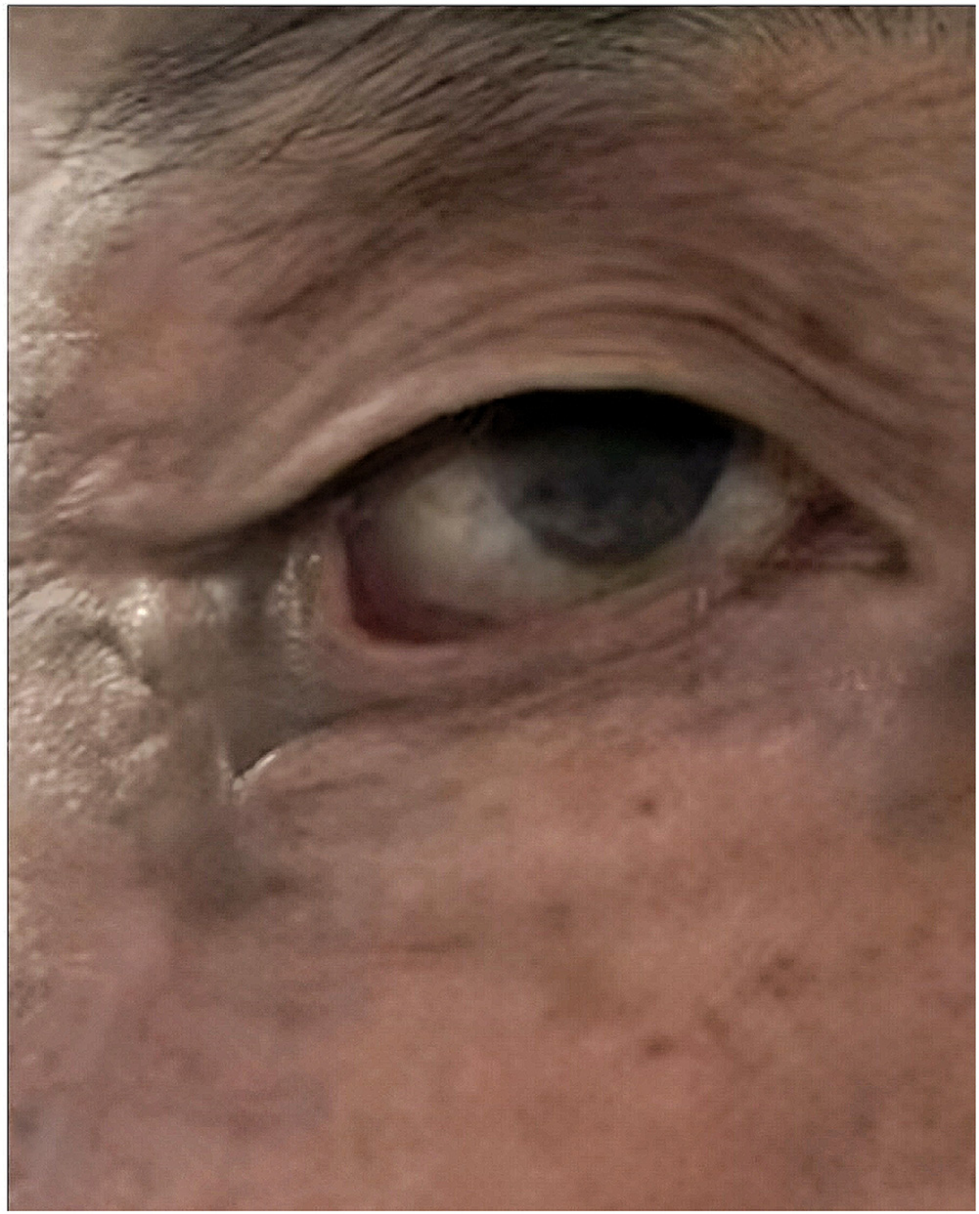
Six-month postoperative follow-up: The ectropion had regressed to the degree seen in.

Physical examination revealed incomplete right eyelid closure, significant eversion of the lower eyelid margin, exposed and congested lower eyelid conjunctiva, and a 2 × 1 cm circular scar at the lateral aspect of the right zygomatic arch, darker than the surrounding skin. The contracted scar produced two additional skin folds compared to, though the uppermost folds in preexisted and were unrelated to the wound healing. Given the patient’s concerns about a patch-like appearance from skin grafting, alternative treatments were discussed. Ultimately, the V-Y advancement flap technique was selected . At a six-month follow-up, only mild residual lower eyelid ectropion persisted (Figure 2). The degree of ectropion matched the initial presentation, but the severe folds had resolved. We speculate that the patient’s excessive scar contracture contributed to impaired wound healing. Residual mild ectropion was attributed to the combined effect of unaddressed horizontal lid laxity and persistent traction from the pre-existing primary scar. Although a second-stage lateral tarsal strip (LTS) procedure was suggested to optimize the outcome, the patient declined further intervention at that time, expressing satisfaction with the significant functional and aesthetic improvement achieved.

Residual mild ectropion was attributed to the combined effect of unaddressed horizontal lid laxity and persistent traction from the pre-existing primary scar. Although a second-stage lateral tarsal strip (LTS) procedure was suggested to optimize the outcome, the patient declined further intervention at that time, expressing satisfaction with the significant functional and aesthetic improvement achieved.

## Surgical procedure

### Anesthesia

A 1:1 mixture of 2 % lidocaine and 0.75 % bupivacaine (with 1:100,000 epinephrine) was administered via subcutaneous infiltration around the scar tissue to ensure uniform anesthesia.

### Incision design and flap creation

A V-shaped skin incision, with its base at the eyelid margin, was made at the most prominent site of the lower eyelid ectropion. The height of the designed V-flap was greater than 2.5 times the height of the ectropion arc to facilitate tension-free advancement. Subdermal dissection was performed to create the V-shaped flap, with meticulous care to preserve surrounding tissues and minimize trauma.

### Flap advancement and fixation

The V-shaped flap was advanced toward the eyelid margin to correct the ectropion . Subcutaneous dissection beneath the incision created sufficient space for suturing. The flap was secured with 4-0 absorbable sutures in a Y-shaped configuration for structural support. Additional 5-0 nonabsorbable interrupted sutures ensured proper repositioning and stabilization of the lower eyelid. The longer the main stem of the Y-shaped suture, the greater the transverse tension, which alleviates more ectropion. Surgeons may adjust the suture length based on individual patient needs.

### Postoperative management

A pressure dressing was applied to minimize bleeding, edema, and promote wound healing.

## Discussion

Cicatricial ectropion often results from excessive scar tension pulling the lower eyelid outward. Three common approaches are described in the literature^1^: Flap techniques (e.g., large advancement flaps^1^, ^2^, ^3^, ^4^) redistribute tension perpendicular to the eyelid margin to counteract scar traction. Full-thickness skin grafts (FTSG) use donor skin (e.g., postauricular) to cover defects after scar release, reducing contracture-related tension.^1^Lateral tarsal strip (LTS) shortens and fixes the lower eyelid tarsus to periosteum to generate tension parallel to the eyelid margin.^5^ These methods may be combined depending on clinical context.^6^

This case represents the first reported use of the V-Y advancement flap for severe cicatricial ectropion. Remarkably effective despite prior use in mild cases,^7^^,^^8^ this technique avoids patch-like appearances from distant flaps/grafts, minimizes muscle dissection, and reduces iatrogenic risks. Its simplicity, reduced suturing, and shorter operative time are notable advantages. Residual ectropion suggests potential for secondary procedures (e.g., LTS). When selecting a surgical technique for cicatricial ectropion, it is crucial to weigh conventional options against patient-specific factors and the primary pathophysiologic cause. Full-thickness skin grafts (FTSG) offer abundant tissue for large defects but carry risks of donor-site morbidity, patch-like appearance, and color mismatch.^1^^,^^6^ The lateral tarsal strip (LTS) procedure effectively addresses horizontal lid laxity but does not primarily correct vertical skin deficiency.^5^ In this case, the patient's strong aversion to skin grafting and the predominant issue of vertical tension from scar contracture made the V-Y advancement flap a particularly suitable choice. This technique effectively redistributes vertical tension without the need for a distant donor site, thereby avoiding a patch-like appearance and better meeting the patient's aesthetic expectations. It is important to note that residual mild ectropion in our patient may indicate an element of unaddressed horizontal laxity, suggesting that a combination with a procedure like LTS could be considered for optimal results in similar cases in the future. Our case illustrates the importance of a tailored, sometimes staged, surgical plan that addresses all layers of pathology while prioritizing flap safety and patient satisfaction. In summary, the V-Y advancement flap is a promising option for cicatricial ectropion, underscoring the need for individualized treatment plans.

## Funding

None.

## Ethical approval

Not required. This case report was conducted in accordance with the principles of the Declaration of Helsinki. Written informed consent was obtained from the patient for publication of this report and accompanying images.

## Declaration of competing interest

None declared.
